# Supplementary material for: Diffusion-synthesized Chest X-rays improve fairness and diagnostic performance
Source: PLOS Digit Health. 2026 Apr 3;5(4):e0001277. doi: 10.1371/journal.pdig.0001277 (PMC13048414; doi:10.1371/journal.pdig.0001277)
Supplement: S1 File — LoRA Ranks Ablation Study. Effect of varying LoRA rank and target modules on generation quality and efficiency. (PDF) [file pdig.0001277.s001.pdf]

Supporting Information: Diffusion-Synthesized Chest X-rays Improve Fairness and Diagnostic Performance

Additional Implementation Details and Algorithm

We provide additional implementation details for fine-tuning Stable Diffusion with LoRA on the CheXpert dataset.

Listing 1: Generating Text Captions from CheXpert Labels

```
conditions = [
    ‘‘No Finding’’, ‘‘Enlarged Cardiomediastinum’’, ‘‘Cardiomegaly’’,
    ‘‘Lung Opacity’’, ‘‘Lung Lesion’’, ‘‘Edema’’, ‘‘Consolidation’’,
    ‘‘Pneumonia’’, ‘‘Atelectasis’’, ‘‘Pneumothorax’’, ‘‘Pleural Effusion’’,
    ‘‘Pleural Other’’, ‘‘Fracture’’, ‘‘Support Devices’’
]

captions = []
for image in images:
    findings = []
    for condition in conditions:
        if image[condition] == 1:
            findings.append(condition)

    caption = "Chest X-ray showing " + ", ".join(findings) \
        if findings else "Normal chest X-ray with no significant findings"
    captions.append(caption)
```

Listing 2: LoRA Configuration for Stable Diffusion U-Net

```
# Inject LoRA into cross-attention layers of U-Net
from peft import get_peft_model, LoraConfig, TaskType
```

```
lora_config = LoraConfig(
    r=4,                        # rank of the LoRA update matrices
    lora_alpha=16,              # scaling factor
    lora_dropout=0.1,           # dropout applied to LoRA weights
    bias="none",                # whether to adapt bias terms
    task_type=TaskType.UNET     # specifies where to inject LoRA
)

unet = get_peft_model(unet, lora_config)
unet.train()  # freeze base weights, only train LoRA parameters
```

Algorithm 1: LoRA Fine-Tuning of Stable Diffusion

```
Input:
    - Pretrained Stable Diffusion components: unet, vae, textEncoder,
      tokenizer, noiseScheduler
    - LoRA applied to U-Net attention layers
    - CheXpert dataloader with (image, caption) pairs
    - Optimizer

for each batch in dataloader do
    input_ids = tokenizer(captions)
    encoder_hidden_states = textEncoder(input_ids)

    latents = vae.encode(batch["image"])  # Convert image to latent space
    noise = sample_random_noise()
    timesteps = sample_random_timesteps()

    noisy_latents = noiseScheduler.add_noise(latents, noise, timesteps)

    noise_pred = unet(noisy_latents, timesteps, encoder_hidden_states)

    loss = mse_loss(noise_pred, noise)
    loss.backward()
    optimizer.step()
    optimizer.zero_grad()
end for
```
