# Supplementary material for: Diffusion-synthesized Chest X-rays improve fairness and diagnostic performance
Source: PLOS Digit Health. 2026 Apr 3;5(4):e0001277. doi: 10.1371/journal.pdig.0001277 (PMC13048414; doi:10.1371/journal.pdig.0001277)
Supplement: S1 Table — Effect of varying LoRA rank and target modules on generation quality and efficiency. (PDF) [file pdig.0001277.s002.pdf]

S1_Table. LoRA Rank–Quality Trade-off. Effect of varying LoRA rank on image generation quality and efficiency. All experiments use target modules `to_q`, `to_k`, and `to_v`. Values are mean ± standard deviation over three runs. Lower FID/KID and higher SSIM/PSNR indicate better image quality.

**LoRA Rank (r)**	**FID** ↓	**KID** ↓	**SSIM** ↑	**PSNR (dB)** ↑
2, 8	52.6 ± 2.1	0.041 ± 0.003	0.812 ± 0.010	21.4 ± 0.3
4, 16	**29.7 ± 1.3**	**0.026 ± 0.002**	0.857 ± 0.006	**23.9 ± 0.2**
16, 32	28.9 ± 1.4	0.025 ± 0.002	**0.861 ± 0.005**	23.6 ± 0.3

Table notes: Increasing the LoRA rank improves reconstruction fidelity but increases computational cost. Moderate ranks (e.g., $r = 4/16$) achieve an optimal trade-off between quality and efficiency. Variability (± SD) confirms consistent trends across runs, supporting robustness of rank selection.
