# Supplementary material for: Diffusion-synthesized Chest X-rays improve fairness and diagnostic performance
Source: PLOS Digit Health. 2026 Apr 3;5(4):e0001277. doi: 10.1371/journal.pdig.0001277 (PMC13048414; doi:10.1371/journal.pdig.0001277)
Supplement: S2 Table — Metrics include Dice Similarity Coefficient (DSC), Intersection over Union (IoU), Structural Similarity Index (SSIM), and Peak Signal-to-Noise Ratio (PSNR). Higher values indicate better focus quality and attention map fidelity. (PDF) [file pdig.0001277.s003.pdf]

S2_Table. Quantitative evaluation of model attention and focus quality using standard metrics. Metrics include Dice Similarity Coefficient (DSC), Intersection over Union (IoU), Structural Similarity Index (SSIM), and Peak Signal-to-Noise Ratio (PSNR). Higher values indicate better focus quality and attention map fidelity.

**Model Variant**	**Training Data**	**DSC**	**IoU**	**SSIM**	**PSNR**
CXR-CLIP$_{\text{Res50}}$	Real	0.85	0.75	0.90	35.2
CXR-CLIP$_{\text{Res50}}$	Synthetic	**0.88**	**0.78**	**0.92**	**36.5**
CXR-CLIP$_{\text{Swin-T}}$	Real	0.83	0.72	0.88	34.8
CXR-CLIP$_{\text{Swin-T}}$	Synthetic	**0.87**	**0.76**	**0.91**	**36.0**
