# Supplementary material for: Diffusion-synthesized Chest X-rays improve fairness and diagnostic performance
Source: PLOS Digit Health. 2026 Apr 3;5(4):e0001277. doi: 10.1371/journal.pdig.0001277 (PMC13048414; doi:10.1371/journal.pdig.0001277)
Supplement: S3 Table — Effect of synthetic data proportion on performance and fairness. Performance (AUC) and demographic fairness (average difference across groups) are shown for Res50 and Swin-T models. (PDF) [file pdig.0001277.s004.pdf]

S3_Table. Effect of synthetic data proportion on classification performance and fairness. AUC and ECE present average classification performance for all diseases of each model, and demographic fairness (average difference across groups) for Res50 and Swin-T models.

Proportion	Model	AUC	ECE	Gender Gap	Race Gap	Age Gap
0%	Res50	0.821	0.036	0.030	0.062	0.058
25%	Res50	0.848	0.032	0.022	0.045	0.042
50%	Res50	0.875	0.030	0.015	0.030	0.028
75%	Res50	0.880	0.039	0.008	0.018	0.015
100%	Res50	0.902	0.030	0.003	0.003	0.005
0%	Swin-T	0.834	0.043	0.032	0.064	0.060
25%	Swin-T	0.850	0.033	0.023	0.046	0.043
50%	Swin-T	0.866	0.036	0.015	0.031	0.028
75%	Swin-T	0.871	0.030	0.009	0.020	0.017
100%	Swin-T	0.899	0.029	0.003	0.002	0.006
