# Supplementary material for: Diffusion-synthesized Chest X-rays improve fairness and diagnostic performance
Source: PLOS Digit Health. 2026 Apr 3;5(4):e0001277. doi: 10.1371/journal.pdig.0001277 (PMC13048414; doi:10.1371/journal.pdig.0001277)
Supplement: S1 Fig — (PDF) [file pdig.0001277.s005.pdf]

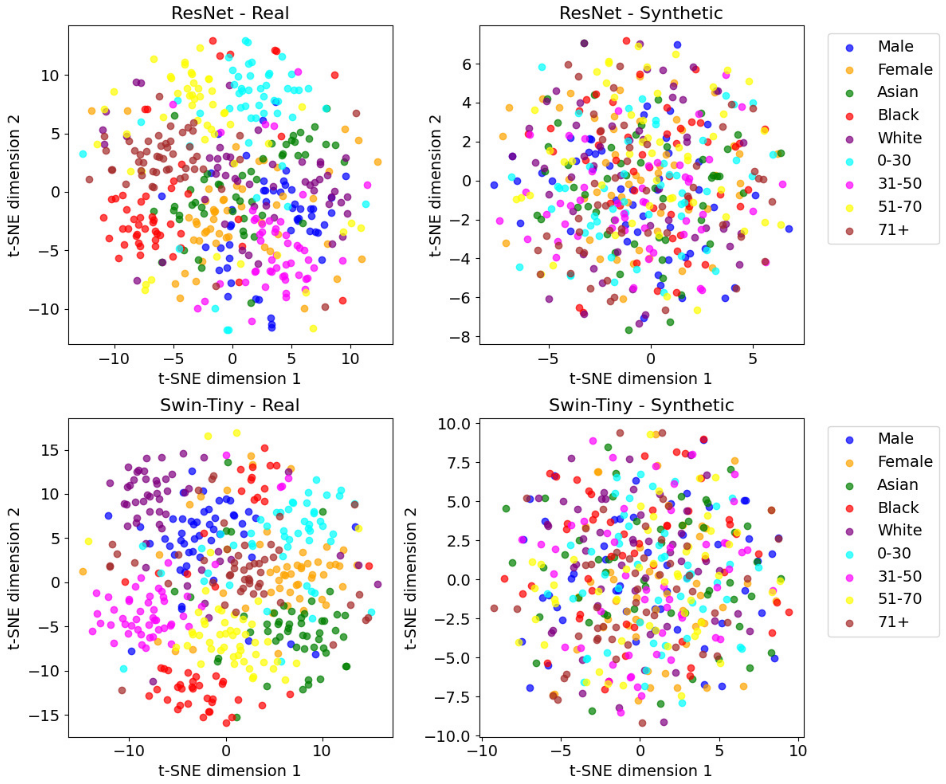


**S1_Fig.** t-SNE of latent embeddings for ResNet (top) and Swin-Tiny (lower). Left: real data, showing distinct demographic clusters; Right: synthetic data, showing overlapping clusters. Synthetic data reduces demographic signals, helping mitigate shortcut learning.
