# Supplementary material for: Diffusion-synthesized Chest X-rays improve fairness and diagnostic performance
Source: PLOS Digit Health. 2026 Apr 3;5(4):e0001277. doi: 10.1371/journal.pdig.0001277 (PMC13048414; doi:10.1371/journal.pdig.0001277)
Supplement: S2 Fig — (PDF) [file pdig.0001277.s006.pdf]

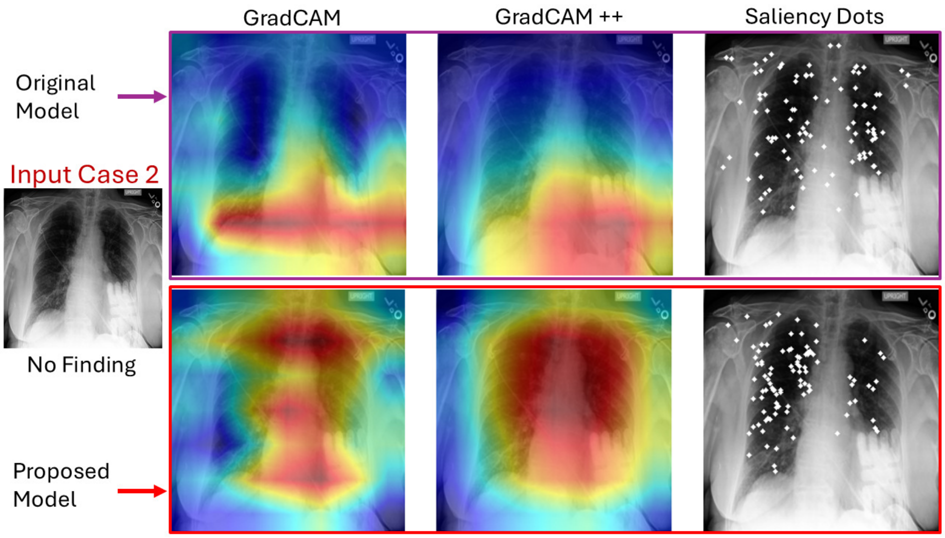

S2_Fig. This figure presents model focus observation for the no finding case, which means the input CXR does not contain any disease information. We can see that the baseline model does not focus on the lung region; however, the proposed model has developed the ability to keep focus on the lung region.
