# Supplementary material for: Diffusion-synthesized Chest X-rays improve fairness and diagnostic performance
Source: PLOS Digit Health. 2026 Apr 3;5(4):e0001277. doi: 10.1371/journal.pdig.0001277 (PMC13048414; doi:10.1371/journal.pdig.0001277)
Supplement: S3 Fig — (PDF) [file pdig.0001277.s007.pdf]

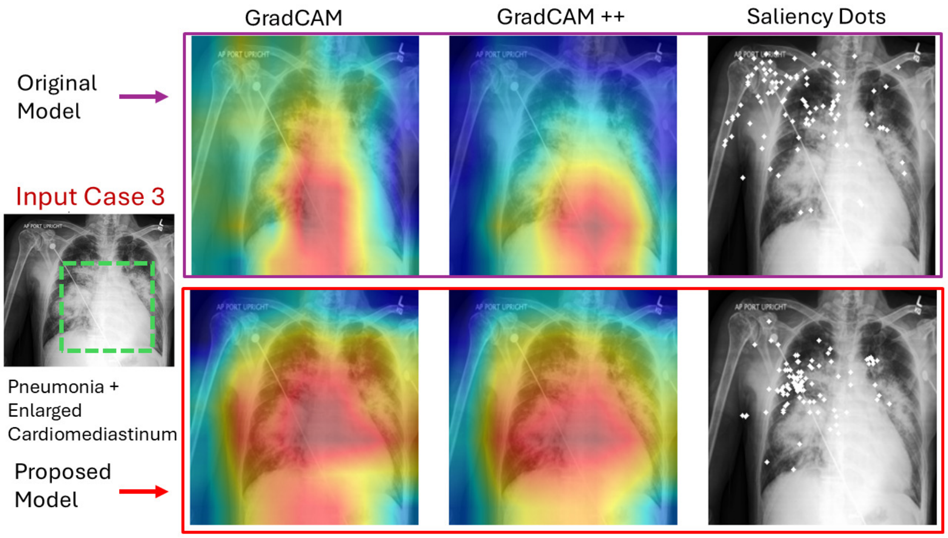

S3_Fig. In this figure, we see that the model takes as input a CXR with pneumonia and an enlarged cardiomediastinum; the baseline model trained on real data struggles to focus on disease areas. However, the proposed model trained on synthetic data keeps focus on disease areas in the lung regions and does not lose confidence regarding demographics.
