# Supplementary material for: Diffusion-synthesized Chest X-rays improve fairness and diagnostic performance
Source: PLOS Digit Health. 2026 Apr 3;5(4):e0001277. doi: 10.1371/journal.pdig.0001277 (PMC13048414; doi:10.1371/journal.pdig.0001277)
Supplement: S4 Fig — (PDF) [file pdig.0001277.s008.pdf]

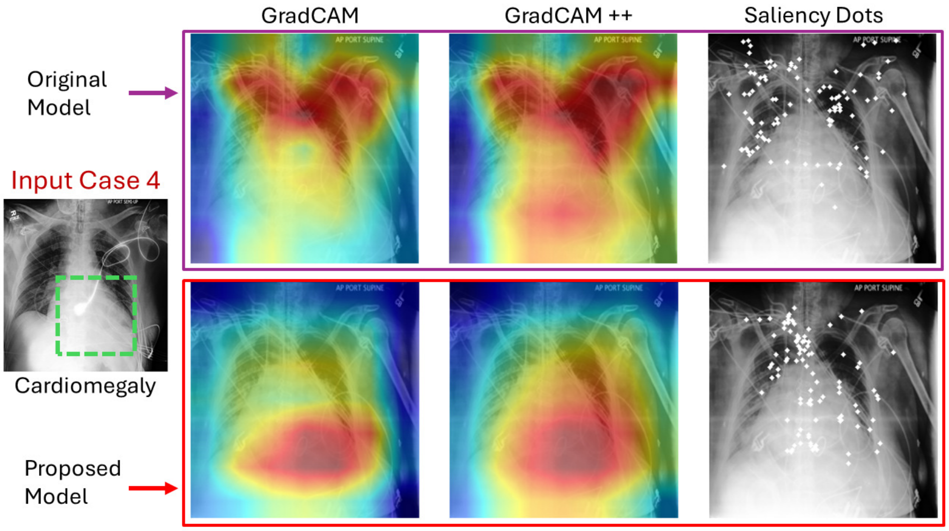

S4_Fig. Cardiomegaly case study model focuses on observation and comparison of the baseline versus the proposed model. A clear difference in the model's focus of observation could be understood.
