# Supplementary material for: Diffusion-synthesized Chest X-rays improve fairness and diagnostic performance
Source: PLOS Digit Health. 2026 Apr 3;5(4):e0001277. doi: 10.1371/journal.pdig.0001277 (PMC13048414; doi:10.1371/journal.pdig.0001277)
Supplement: S5 Fig — (PDF) [file pdig.0001277.s009.pdf]

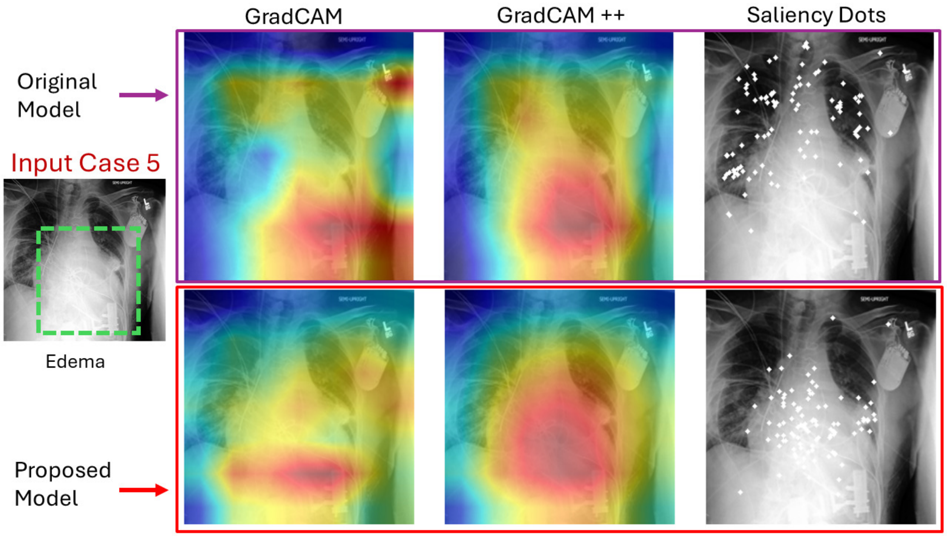

S5_Fig. This figure presents model focus observation for the edema disease from a hard CXR classification category. We can see that the baseline model does not focus on the lung region; however, the proposed model has developed the ability to keep focus on edema disease.
