# Supplementary material for: Diffusion-synthesized Chest X-rays improve fairness and diagnostic performance
Source: PLOS Digit Health. 2026 Apr 3;5(4):e0001277. doi: 10.1371/journal.pdig.0001277 (PMC13048414; doi:10.1371/journal.pdig.0001277)
Supplement: S6 Fig — (PDF) [file pdig.0001277.s010.pdf]

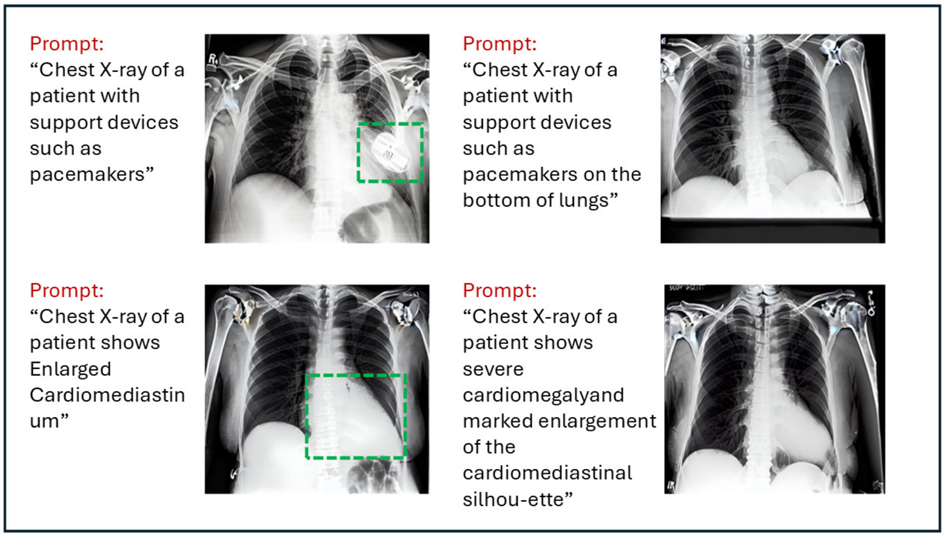

S6_Fig. The figure represents the synthetic CXRs with different prompts. In the top row on the left for both rows, we can see that the model works perfectly with the specific prompts, according to the given information. However, when we give detailed prompts for a specific location or direction, the model struggles to generate such CXRs.
